# Association between brain-derived neurotrophic factor gene polymorphisms and fibromyalgia in a Korean population: a multicenter study

**DOI:** 10.1186/s13075-018-1726-5

**Published:** 2018-10-03

**Authors:** Dong-Jin Park, Seong-Ho Kim, Seong-Su Nah, Ji Hyun Lee, Seong-Kyu Kim, Yeon-Ah Lee, Seung-Jae Hong, Hyun-Sook Kim, Hye-Soon Lee, Hyoun Ah Kim, Chung-Il Joung, Sang-Hyon Kim, Shin-Seok Lee

**Affiliations:** 1 Division of Rheumatology, Department of Internal Medicine, Chonnam National University Medical School and Hospital, 42 Jebong-ro, Dong-gu, Gwangju, 61469 Republic of Korea; 2 Department of Internal Medicine, Inje University Haeundae Paik Hospital, Busan, Korea; 3 Department of Internal Medicine, Soonchunhyang University, College of Medicine, Cheonan, Korea; 4 Department of Internal Medicine, Maryknoll Medical Center, Busan, Korea; 5 Department of Internal Medicine, Catholic University of Daegu, School of Medicine, Daegu, Korea; 6 Department of Internal Medicine, School of Medicine, Kyung Hee University, Seoul, Korea; 7 Department of Internal Medicine, Soonchunhyang University Seoul Hospital, Seoul, Korea; 8 Hanyang University College of Medicine and the Hospital for Rheumatic Diseases, Seoul, Korea; 9 Department of Allergy and Rheumatology, Ajou University Hospital, Ajou University School of Medicine, Suwon, Korea; 10 Department of Internal Medicine, Konyang University Medical School, Daejeon, Korea; 11 Departments of Internal Medicine, School of Medicine, Keimyung University, Daegu, Korea

**Keywords:** Fibromyalgia, Brain-derived neurotrophic factor, Genetics, Polymorphism

## Abstract

**Background:**

Several lines of evidence imply that brain-derived neurotrophic factor (BDNF) is involved in the pathophysiology of fibromyalgia (FM); in this regard, patients with FM have altered blood and cerebrospinal fluid levels of BDNF. In this study, we explored the association between *BDNF* gene polymorphisms and FM susceptibility and the severity of symptoms.

**Methods:**

In total, 409 patients with FM and 423 healthy controls in 10 medical centers were enrolled from the Korean nationwide FM survey. The alleles and genotypes at 10 positions in the *BDNF* gene were genotyped.

**Results:**

The allele and genotype frequencies of *BDNF* rs11030104 differed significantly between the patients with FM and the controls (*P* = 0.031). The GG genotype of rs11030104 had a protective effect against FM (*P* = 0.016), and the G allele of rs11030104 was negatively associated with the presence of FM compared with the A allele (*P* = 0.013). In comparison, although the allele and genotype frequencies of *BDNF* rs12273539 did not differ between the two groups, the TT genotype of *BDNF* rs12273539 was associated with susceptibility to FM (*P* = 0.038). Haplotype analyses implied that some *BDNF* haplotypes have a protective effect against FM. Finally, several genotypes and haplotypes of the *BDNF* gene contributed to specific symptoms of FM.

**Conclusions:**

This study is the first to evaluate the associations between *BDNF* gene polymorphisms and FM. Our results imply that some *BDNF* single-nucleotide polymorphisms and haplotypes are associated with susceptibility to, and contribute to the symptoms of, FM.

## Background

Fibromyalgia (FM) is a common rheumatic syndrome characterized by chronic widespread pain, and is often accompanied by diverse symptoms including fatigue, sleep disorders, memory loss, joint stiffness, and affective distress [1]. The prevalence of FM in the general population is reportedly 1–5%, and it is more prevalent among women than men [2]. Although its pathogenesis is unclear, FM is recognized as an outcome of the interactions of multiple genetic, psychological, neurobiological, and environmental factors [3].

The familial aggregation observed among patients with FM implies that genetic factors are important contributors to the etiology of FM [4]. Recent genetic studies have advanced our understanding of the pathogenesis of FM. These studies have shown that certain gene polymorphisms alter pain sensitivity and increase susceptibility to FM [5]. In particular, polymorphisms of genes involved in the pain transmission pathway, such as the serotoninergic, dopaminergic, and catecholaminergic systems, have received much attention as possible genetic factors in FM [6, 7]. However, those genetic factors do not fully account for the pathophysiology and symptoms of FM. Therefore, efforts to identify other genetic factors that contribute to FM are ongoing.

Brain-derived neurotrophic factor (BDNF) is involved in neuronal survival, growth, and differentiation during development of the central and peripheral nervous systems [8]. BDNF is important in the transmission of physiologic or pathologic pain [9]. BDNF is responsible for modulation of nociceptive inputs and enhanced hyperalgesia by a N-methyl-D-aspartate (NMDA) receptor-mediated mechanism [10]. Moreover, dysregulation of BDNF in the dorsal root ganglion (DRG) and spinal cord contributes to chronic pain hypersensitivity [11]. In addition, several lines of evidence have converged to imply that BDNF is involved in the pathophysiology of FM. Indeed, patients with FM have been shown to have altered serum and plasma levels of BDNF compared to healthy controls [12–14].

However, whether polymorphisms of the *BDNF* gene are associated with FM remains an open question. The objective of this study was to evaluate the associations between *BDNF* gene polymorphisms and FM susceptibility and clinical symptoms, using a large population of ethnically homogenous Koreans.

## Methods

### Study design and population

We performed a multicenter, nationwide FM cohort study (the Korean Nationwide FM Survey) in the Korean population. In the Korean Nationwide FM Survey, we established a prospective cohort to evaluate the pathophysiology of FM, and the clinical manifestations and outcomes of Korean patients with FM. The study participants were recruited from the outpatient rheumatology clinics of 10 medical centers. In this study, a cross-sectional design was employed to evaluate the association between *BDNF* gene polymorphisms and susceptibility to, and symptom severity of, FM. As reported previously [15], we enrolled 409 patients with FM (382 women and 27 men) with a mean (SD) age of 48.1 (10.9) years. At the time of the initial diagnosis, patients with FM were diagnosed according to the classification criteria for FM proposed by the American College of Rheumatology (ACR) in 1990 [1]. The mean (SD) symptom duration before diagnosis was 8.5 (8.3) years, and the mean (SD) disease duration after initial diagnosis was 1.9 (3.0) years. Based on health surveys for chronic pain, we recruited 423 healthy controls (397 women, 25 men) with a mean (SD) age of 45.5 (12.5) years and no history of chronic pain, including FM. Healthy controls were recruited randomly, without matching for age or sex, among the individuals visiting the general health examination clinics at each medical center. This research complied with the Helsinki Declaration, and written informed consent was obtained from all participants at the time of recruitment. Exactly the same informed consult form (ICF) and study protocol were provided to the independent Institutional Review Board/Ethics Committee (IRB/EC) at each medical center, and each IRB/EC reviewed the appropriateness of the protocol and risks and benefits to the study participants. Ultimately, the IRB/EC at each medical center independently approved this study without revision of the ICF or study protocol.

### Procedures

The patients with FM were interviewed at the time of enrollment to determine their demographics and clinical characteristics, including age, sex, body mass index, and symptom and disease duration. In addition, at enrollment, peripheral venous blood was sampled and then stored in an ethylenediaminetetraacetate (EDTA)-coated tube. Tender points were assessed by thumb palpation according to the standardized tender point survey protocol [16]. The number of tender points was assessed at 18 sites on the body. The intensity at each tender point was assessed by determining the tender point score as follows: 0, no tenderness; 1, light tenderness (confirming answer when asked); 2, moderate tenderness (spontaneous verbal response); and 3, severe tenderness (moving away). Therefore, the number of tender points ranged from 0 to 18, and the possible total scores of the tender points ranged from 0 to 54. Furthermore, extensive clinical assessments of patients with FM enrolled in the cohort were undertaken using a self-report questionnaire and semi-structured questionnaires. The Korean version of the Fibromyalgia Impact Questionnaire (FIQ) was used to assess the functional abilities and severity of FM [17], and the Brief Fatigue Inventory (BFI) and the Beck Depression Inventory (BDI) were used to evaluate the severity of fatigue and depression, respectively [18, 19]. The 36-item Medical Outcomes Study Short-Form Health Survey (SF-36) was used to access the quality of life of the patients with FM [20]. In addition, we also evaluated the severity of anxiety using the State-Trait Anxiety Inventory (STAI)-I and STAI-II [21].

The patients had been treated with standard medications for FM, based on the clinical judgment of their attending rheumatologist. Concomitant medications, used at the time of enrollment, included tricyclic antidepressants (TCA), selective serotonin reuptake inhibitors (SSRI), serotonin-norepinephrine reuptake inhibitors (SNRI), pregabalin, gabapentin, nonsteroidal anti-inflammatory drugs (NSAIDs), acetaminophen, benzodiazepine, tramadol, and muscle relaxants.

### Genotyping of *BDNF* polymorphisms

The assay reagents for rs2883187(C > T), rs7103873 (G > C), rs7103411(C > T), rs10835210(C > A), rs11030104 (A > G), rs12273539(C > T), rs11030102(C > G), rs11030101(A > T), rs6265(G > A) and rs7124442(C > T) in the *BDNF* gene were designed by Applied Biosystems (Applied Biosystems). The reagents consisted of TaqMan MGB probes (FAM and VIC dye-labeled). Each reaction (10 μL) comprised 0.125 μL of 40X reagents, 5 μL of 2X TaqMan Genotyping Master Mix (Applied Biosystems) and 2 μL of 50 ng genomic DNA. The PCR conditions were 1 cycle at 95 °C for 10 min, followed by 40 cycles at 95 °C for 15 s and 60 °C for 1 min. The PCR reactions were performed using an ABI plus instrument (Applied Biosystems). The samples were read and analyzed using ABI plus software (Applied Biosystems). The sequences of the primers used for TaqMan probe genotyping of the *BDNF* gene are summarized in Table 1.

**Table 1:** Primer sequences used for TaqMan probe genotyping of *BDNF*

Regions	Primers	Primer sequence (5′ → 3′)
rs 2883187	Forward	GTGAGGCATCCGGCCCGGCTGGGGA
	Reverse	CGGAGCGCGGTCTCGGCAGCTCCCC
rs 7103873	Forward	AGGACCTTTTACCCCCAAATGTAGA
	Reverse	ACTAAATGAAAAACCATTCTTTAAA
rs 7103411	Forward	GGAGCGCACTGTAAAGATACTGATA
	Reverse	GAACACGAATGTGAGATCAATGTTG
rs 10835210	Forward	CTTAACTGTAAAGCACAGGAAAGTG
	Reverse	TCATTACTTGTAGCTTAATGCAGGA
rs 11030104	Forward	ATTAAAAAGCAGATAACACTACCAC
	Reverse	TACTAACTGTCCTACAATTTCCTGT
rs 12273539	Forward	ACTCAATGCTTCATCACTTCTGCTC
	Reverse	GATCAGGACAGAGTCCTTGGAGTGC
rs 11030102	Forward	CTACTTCTCAGTTCTGAGGCATGGA
	Reverse	TTACAAAAAGACACATACATGCAAT
rs 11030101	Forward	GATACTCTATTATAGCAAAGAAGAA
	Reverse	GATAATTTCATTGAGCCATCCTGTT
rs 6265	Forward	TCCTCATCCAACAGCTCTTCTATCA
	Reverse	GTGTTCGAAAGTGTCAGCCAATGAT
rs 7124442	Forward	AAGGAAGCTGCATAAAGTTGACATA
	Reverse	AGCAGATATTCCAAGCATTCCTTAC

*BDNF* brain-derived neurotrophic factor

### Statistical analysis

Statistical analyses were performed using IBM SPSS statistics (SPSS version 21; IBM SPSS Inc., Chicago, IL, USA). *P* values <0.05 were considered to indicate statistical significance. Each *BDNF* gene polymorphism was tested for Hardy-Weinberg equilibrium. The genotype and haplotype frequencies of the *BDNF* single-nucleotide polymorphisms (SNPs) were compared between the patients with FM and controls by Fisher’s exact test or Pearson’s chi-squared test. The association between each *BDNF* genotype and haplotype and susceptibility to FM was defined by logistic regression analysis. Analysis of covariance (ANCOVA), adjusted for age and sex, was used to explore the differences in the clinical measurements of the patients with FM according to *BDNF* genotype and haplotype. Haplotype structures were constructed and their frequencies estimated by combined allele analysis using PHASE v2.1.1 software (Department of Statistics, University of Washington, Seattle, WA, USA). We carried out a permutation test for the null hypothesis that the patients with FM and the healthy controls are random draws from a common set of haplotype frequencies (number of permutations performed = 10,000).

## Results

### BDNF genotypes and alleles and their association with clinical measurements

The *BDNF* SNPs were successfully genotyped in all enrolled subjects, except for 5 controls with *BDNF* rs2883187, 1 patient and 16 controls with *BDNF* rs7103873, 2 controls with *BDNF* rs7103411, 1 patient and 10 controls with *BDNF* rs10835210, 2 patients and 3 controls with *BDNF* rs11030104, 1 control with *BDNF* rs12273539, 1 patient and 1 control with *BDNF* rs11030102, 1 patient and 3 controls with *BDNF* rs11030101, 1 patient and 4 controls with *BDNF* rs6265, and 2 patients and 2 controls with *BDNF* rs7124442. The genotype distributions of the *BDNF* SNPs were consistent with Hardy-Weinberg equilibrium in both the patients and controls.

Among the *BDNF* SNPs, the allele and genotype frequencies of *BDNF* SNP rs11030104 were significantly different between the patients with FM and controls. Furthermore, patients with the GG genotype of rs11030104 were found less frequently in patients with FM after adjusting for age and sex (OR 0.619; 95% confidence interval (CI) 0.419–0.0913; *P* = 0.016). In addition, the G allele was negatively associated with the presence of FM compared to the A allele (OR = 0.781, 95% CI 0.641–0.950, *P* = 0.013). In comparison, although the allele and genotype frequencies of the SNPs of *BDNF* rs12273539 were not significantly different between the patients with FM and controls, the TT genotype of rs12273539 was found more frequently in patients with FM in the age-adjusted and sex-adjusted model (OR 2.586; 95% CI 1.052–6.360; *P* = 0.038) (Table 2).

**Table 2:** Genotype and allele analyses of *BDNF* in patients with fibromyalgia and healthy controls^a^

Marker	Genotype/allele	Contol, *n* (%)	Fibromyalgia, *n* (%)	Exact *p* value^b^	OR (95% CI), *p* value^c^	OR (95% CI), *p* value, adjusted by age, sex^‡^
rs2883187	C/C	115 (27.5)	100 (24.4)	0.218	1	1
	C/T	220 (52.6)	208 (50.9)		1.087 (0.783–1.510), *p* = 0.617	1.044 (0.747–1.458), *p* = 0.802
	T/T	83 (19.9)	101 (24.7)		1.399 (0.943–2.078), *p* = 0.096	1.340 (0.897–2.002), *p* = 0.152
	C	450 (53.8)	408 (49.9)	0.119	1	
	T	386 (46.2)	410 (50.1)		1.172 (0.966–1.421), *p* = 0.108	1.147 (0.943–1.395), *p* = 0.171
rs7103873	G/G	113 (27.8)	98 (24.0)	0.245	1	1
	C/G	210 (51.6)	208 (51.0)		1.142 (0.820–1.591), *p* = 0.432	1.110 (0.791–1.556), *p* = 0.546
	C/C	84 (20.6)	102 (25.0)		1.400 (0.943–2.080), *p* = 0.095	1.345 (0.899–2.010), *p* = 0.149
	G	436 (53.6)	404 (49.5)	0.112	1	
	C	378 (46.4)	412 (50.5)		1.176 (0.968–1.429), *p* = 0.102	1.153 (0.946–1.405), *p* = 0.158
rs7103411	C/C	120 (28.5)	128 (31.3)	0.638	1	1
	C/T	208 (49.4)	198 (48.4)		0.892 (0.651–1.224), *p* = 0.48	0.884 (0.641–1.220), *p* = 0.454
	T/T	93 (22.1)	83 (20.3)		0.837 (0.568–1.232), *p* = 0.366	0.865 (0.584–1.280), *p* = 0.468
	C	448 (53.2)	454 (55.5)	0.374	1	
	T	394 (46.8)	364 (44.5)		0.912 (0.751–1.106), *p* = 0.348	0.925 (0.76–1.125), *p* = 0.435
rs10835210	C/C	204 (49.4)	196 (48.0)	0.725	1	1
	A/C	175 (42.4)	172 (42.2)		1.023 (0.767–1.364), *p* = 0.877	0.991 (0.740–1.326), *p* = 0.949
	A/A	34 (8.2)	40 (9.8)		1.224 (0.745–2.014), *p* = 0.425	1.183 (0.714–1.957), *p* = 0.514
	C	583 (70.6)	564 (69.1)	0.554	1	
	A	243 (29.4)	252 (30.9)		1.072 (0.868–1.324), *p* = 0.518	1.048 (0.846–1.297), *p* = 0.671
rs11030104	A/A	101 (24.0)	126 (31.0)	0.031	1	1
	A/G	205 (48.8)	196 (48.2)		0.766 (0.553–1.063), *p* = 0.111	0.758 (0.544–1.057), *p* = 0.102
	G/G	114 (27.1)	85 (20.9)		0.598 (0.407–0.877), *p* = 0.009	0.619 (0.419–0.913), *p* = 0.016
	A	407 (48.5)	448 (55.0)	0.009	1	
	G	433 (51.5)	366 (45.0)		0.768 (0.633–0.932), *p* = 0.007	0.781 (0.641–0.95), *p* = 0.013
rs12273539	C/C	283 (67.1)	268 (65.4)	0.101	1	1
	C/T	132 (31.3)	125 (30.5)		1 (0.744–1.345), *p* = 1	1.009 (0.747–1.362), *p* = 0.955
	T/T	7 (1.7)	17 (4.1)		2.564 (1.047–6.282), *p* = 0.039	2.586 (1.052–6.36), *p* = 0.038
	C	698 (82.7)	661 (80.6)	0.299	1	
	T	146 (17.3)	159 (19.4)		1.150 (0.897–1.475), *p* = 0.27	1.161 (0.902–1.493), *p* = 0.246
rs11030102	C/C	419 (99.3)	402 (98.5)	0.334	1	1
	C/G	3 (0.7)	6 (1.5)		2.085 (0.518–8.392), *p* = 0.301	2.129 (0.524–8.649), *p* = 0.291
	C	841 (99.6)	810 (99.3)	0.335	1	
	G	3 (0.4)	6 (0.7)		2.077 (0.518–8.326), *p* = 0.302	2.12 (0.524–8.58), *p* = 0.292
rs11030101	A/A	208 (49.5)	197 (48.3)	0.752	1	1
	A/T	178 (42.4)	172 (42.2)		1.020 (0.766–1.358), *p* = 0.891	0.985 (0.737–1.317), *p* = 0.92
	T/T	34 (8.1)	39 (9.6)		1.211 (0.735–1.996), *p* = 0.452	1.152 (0.694–1.912), *p* = 0.583
	A	594 (70.7)	566 (69.4)	0.585	1	
	T	246 (29.3)	250 (30.6)		1.067 (0.864–1.316), *p* = 0.548	1.036 (0.837–1.283), *p* = 0.743
rs6265	G/G	96 (22.9)	87 (21.3)	0.770	1	1
	A/G	204 (48.7)	197 (48.3)		1.066 (0.751–1.512), *p* = 0.722	1.017 (0.712–1.451), *p* = 0.928
	A/A	119 (28.4)	124 (30.4)		1.150 (0.783–1.688), *p* = 0.476	1.110 (0.752–1.638), *p* = 0.599
	G	396 (47.3)	371 (45.5)	0.496	1	
	A	442 (52.7)	445 (54.5)		1.075 (0.886–1.304), *p* = 0.466	1.058 (0.869–1.287), *p* = 0.575
rs7124442	C/C	2 (0.5)	0 (0)	0.574	1	1
	C/T	51 (12.1)	47 (11.5)		718,117.521 (0-Inf), *p* = 0.972	683,123.831 (0-Inf), *p* = 0.972
	T/T	368 (87.4)	360 (88.5)		762,294.038 (0-Inf), *p* = 0.971	682,163.974 (0-Inf), *p* = 0.972
	C	55 (6.5)	47 (5.8)	0.590	1	
	T	787 (93.5)	767 (94.2)		1.140 (0.763–1.705), *p* = 0.521	1.078 (0.716–1.623), *p* = 0.72

*BDNF* brain-derived neurotrophic factor

^a^Missing data were excluded from the analyses: *BDNF* rs2883187 (5 controls), *BDNF* rs7103873 (1 patient and 16 controls), *BDNF* rs7103411 (2 controls), *BDNF* rs10835210 (1 patient and 10 controls), *BDNF* rs11030104 (2 patients and 3 controls), *BDNF* rs12273539 (1 control), *BDNF* rs11030102 (1 patient and 1 control), *BDNF* rs11030101 (1 patient and 3 controls), *BDNF* rs6265 (4 controls and 1 patient), and *BDNF* rs7124442 (2 patients and 2 controls)

^b^Value was determined by Fisher’s exact test or χ^2^ test

^c^Logistic regression analyses were used to calculate the OR (95% CI; confidence interval)

Within the FM cohort, patients with the CG genotype of *BDNF* rs11030102 had more severe fatigue symptoms (measured by the BFI) and anxiety symptoms (measured by the STAI-I) than did the other genotypes (*P* = 0.001 and *P* = 0.032, respectively). Furthermore, both rs11030101 and rs10835210 were associated with the trait of anxiety (measured by the STAI-II) in patients with FM (*P* = 0.029 and *P* = 0.033, respectively). No associations were observed between clinical measurements and the other *BDNF* SNPs (Table 3).

**Table 3:** Least-squares means (95% CI) of responses in patients with fibromyalgia, according to genotype

Position	Genotype	Number^a^	Tender point number	Tender point count	FIQ	BFI	PCS	MCS	BDI	STAI-I	STAI-II
rs2883187	C/C	100	13.38 (12.21–14.56)	25.17 (21.2–29.14)	58.56 (53.23–63.9)	7.25 (5.28–9.22)	38.02 (35.88–40.16)	31.25 (27.83–34.67)	18.09 (15.05–21.12)	51.56 (48.06–55.06)	51.97 (48.73–55.21)
	C/T	208	13.80 (12.83–14.77)	25.70 (22.43–28.96)	610 (56.69–65.32)	6.27 (4.66–7.89)	37.29 (35.56–39.03)	32.62 (29.85–35.39)	18.57 (16.11–21.02)	49.73 (46.9–52.56)	50.2 (47.59–52.81)
	T/T	101	13.77 (12.54–15.0)	25.85 (21.68–30.03)	58.21 (52.66–63.77)	7.16 (5.11–9.22)	38.17 (35.94–40.41)	34.26 (30.69–37.83)	16.89 (13.7–20.08)	49.49 (45.82–53.15)	48.29 (44.9–51.68)
*p* value^†^			0.739	0.947	0.449	0.466	0.625	0.299	0.54	0.478	0.138
rs7103873	G/G	98	13.37 (12.18–14.55)	25.01 (21.01–29.01)	57.96 (52.56–63.35)	7.34 (5.35–9.32)	37.96 (35.8–40.12)	31.39 (27.93–34.85)	17.78 (14.71–20.85)	51.52 (47.98–55.06)	51.9 (48.63–55.18)
	G/C	208	13.84 (12.88–14.81)	25.97 (22.73–29.21)	60.89 (56.60–65.19)	6.25 (4.65–7.85)	37.34 (35.62–39.07)	32.83 (30.07–35.6)	18.45 (16.01–20.9)	49.51 (46.7–52.32)	50.08 (47.48–52.68)
	C/C	102	13.66 (12.42–14.9)	25.28 (21.08–29.49)	59.04 (53.43–64.64)	7.26 (5.19–9.33)	38.07 (35.82–40.32)	33.52 (29.92–37.13)	17.4 (14.18–20.62)	50.33 (46.64–54.02)	48.72 (45.31–52.13)
*p* value^b^			0.69	0.857	0.479	0.389	0.721	0.523	0.755	0.484	0.224
rs7103411	C/C	128	13.79 (12.68–14.9)	25.97 (22.21–29.72)	60.26 (55.22–65.3)	6.97 (5.11–8.83)	37.76 (35.73–39.79)	32.88 (29.64–36.12)	18.25 (15.37–21.14)	50.48 (47.17–53.8)	49.28 (46.21–52.35)
	C/T	198	13.74 (12.74–14.74)	25.65 (22.27–29.02)	60.54 (56.09–64.99)	6.07 (4.41–7.73)	37.5 (35.71–39.29)	33.0 (30.14–35.87)	18.32 (15.78–20.85)	49.55 (46.62–52.47)	50.44 (47.74–53.15)
	T/T	83	13.46 (12.23–14.68)	25.0 (20.87–29.14)	57.81 (52.24–63.38)	7.64 (5.59–9.68)	37.79 (35.55–40.02)	31.41 (27.84–34.98)	17.5 (14.33–20.67)	50.89 (47.22–54.55)	51.09 (47.69–54.48)
*p* value^b^			0.859	0.907	0.582	0.249	0.947	0.635	0.86	0.705	0.578
rs10835210	C/C	196	13.49 (12.50–14.47)	25.67 (22.37–28.97)	59.70 (55.30–64.10)	6.91 (5.27–8.55)	37.78 (36.02–39.55)	31.77 (28.96–34.58)	18.87 (16.37–21.36)	51.04 (48.18–53.9)	51.77 (49.13–54.41)
	C/A	172	13.87 (12.84–14.91)	25.51 (22.04–28.99)	60.15 (55.46–64.85)	6.66 (4.93–8.4)	37.3 (35.41–39.18)	33.19 (30.18–36.2)	17.17 (14.5–19.83)	48.42 (45.35–51.48)	48.63 (45.81–51.45)
	A/A	40	13.96 (12.18–15.74)	24.85 (18.87–30.83)	59.67 (51.64–67.7)	5.46 (2.52–8.4)	38.45 (35.22–41.67)	35.31 (30.17–40.45)	17.78 (13.17–22.4)	51.79 (46.49–57.08)	47.51 (42.62–52.4)
*p* value^b^			0.675	0.962	0.977	0.609	0.728	0.301	0.384	0.138	0.029
rs11030104	A/A	126	13.80 (12.68–14.92)	25.99 (22.22–29.75)	60.27 (55.2–65.35)	7.0 (5.13–8.87)	37.82 (35.77–39.86)	32.86 (29.6–36.12)	18.33 (15.43–21.23)	50.46 (47.13–53.79)	49.29 (46.2–52.37)
	A/G	196	13.79 (12.79–14.8)	25.67 (22.3–29.03)	60.66 (56.19–65.13)	6.09 (4.42–7.76)	37.4 (35.60–39.20)	32.9 (30.02–35.77)	18.41 (15.87–20.95)	49.69 (46.76–52.61)	50.53 (47.82–53.24)
	G/G	85	13.31 (12.08–14.53)	24.75 (20.64–28.86)	57.55 (51.98–63.12)	7.58 (5.53–9.62)	37.86 (35.63–40.1)	31.6 (28.03–35.16)	17.31 (14.15–20.47)	50.63 (46.99–54.28)	50.88 (47.49–54.27)
*p* value^b^			0.677	0.845	0.498	0.274	0.871	0.731	0.757	0.821	0.616
rs12273539	C/C	268	13.63 (12.7–14.55)	25.24 (22.11–28.37)	59.03 (54.81–63.25)	6.51 (4.95–8.07)	37.67 (35.98–39.37)	33.14 (30.42–35.86)	17.45 (15.05–19.85)	49.9 (47.14–52.66)	49.52 (46.96–52.09)
	C/T	125	14.13 (13.01–15.24)	27.04 (23.26–30.81)	62.11 (57.15–67.08)	6.75 (4.89–8.61)	37.48 (35.48–39.47)	31.56 (28.36–34.76)	19.34 (16.5–22.18)	50.66 (47.4–53.92)	51.73 (48.7–54.75)
	T/T	17	11.90 (9.7–14.1)	21.96 (14.52–29.41)	57.64 (47.31–67.96)	8.92 (5.15–12.69)	38.13 (33.98–42.27)	31.82 (25.17–38.48)	20.22 (14.35–26.09)	49.54 (42.8–56.29)	50.87 (44.61–57.14)
*p* value^b^			0.131	0.319	0.349	0.432	0.945	0.533	0.26	0.862	0.271
rs11030102	C/C	402	13.67 (12.8–14.55)	25.57 (22.61–28.53)	59.83 (55.89–63.77)	6.62 (5.17–8.07)	37.66 (36.07–39.25)	32.67 (30.14–35.21)	18.06 (15.83–20.29)	50.02 (47.45–52.59)	50.16 (47.78–52.55)
	C/G	6	17.58 (13.01–22.15)	36.13 (20.73–51.54)	72.91 (51.53–94.29)	19.07 (11.35–26.79)	35.01 (26.4–43.63)	23.8 (10.04–37.56)	29.58 (17.47–41.69)	65.06 (51.13–78.99)	62.56 (49.62–75.51)
*p* value^b^			0.089	0.172	0.224	0.001	0.542	0.2	0.059	0.032	0.057
rs11030101	A/A	197	13.49 (12.51–14.47)	25.69 (22.38–29.01)	59.82 (55.43–64.22)	6.91 (5.27–8.55)	37.79 (36.02–39.55)	31.73 (28.92–34.54)	18.94 (16.45–21.43)	51.01 (48.15–53.87)	51.77 (49.13–54.42)
	A/T	172	13.84 (12.81–14.87)	25.38 (21.9–28.87)	60.26 (55.57–64.95)	6.69 (4.95–8.43)	37.34 (35.45–39.22)	33.14 (30.14–36.15)	17.1 (14.43–19.76)	48.41 (45.34–51.48)	48.57 (45.75–51.4)
	T/T	39	14.23 (12.45–16.01)	26.37 (20.36–32.37)	58.75 (50.73–66.77)	5.33 (2.39–8.27)	38.16 (34.93–41.39)	35.94 (30.8–41.08)	17.66 (13.12–22.21)	52.15 (46.94–57.37)	48.07 (43.25–52.89)
*p* value^b^			0.605	0.943	0.929	0.555	0.816	0.212	0.325	0.126	0.033
rs6265	G/G	87	13.29 (12.07–14.51)	24.66 (20.55–28.78)	57.67 (52.14–63.21)	7.55 (5.52–9.59)	37.83 (35.61–40.06)	31.56 (28.01–35.11)	17.44 (14.29–20.59)	50.62 (46.97–54.26)	50.94 (47.56–54.32)
	G/A	197	13.77 (12.77–14.77)	25.62 (22.26–28.99)	60.72 (56.27–65.18)	6.11 (4.44–7.77)	37.45 (35.65–39.24)	32.92 (30.05–35.79)	18.27 (15.74–20.81)	49.6 (46.67–52.52)	50.43 (47.71–53.14)
	A/A	124	13.88 (12.76–15)	26.34 (22.56–30.13)	60.17 (55.09–65.24)	6.98 (5.11–8.86)	37.83 (35.79–39.88)	32.91 (29.64–36.18)	18.37 (15.47–21.28)	50.58 (47.24–53.91)	49.37 (46.28–52.46)
*p* value^b^			0.634	0.746	0.513	0.293	0.898	0.702	0.829	0.766	0.657
rs7124442	C/T	47	13.57 (12.12–15.03)	26.18 (21.3–31.07)	62.15 (55.49–68.81)	5.60 (3.16–8.05)	37.37 (34.69–40.05)	30.74 (26.46–35.03)	19.98 (16.2–23.76)	50.67 (46.27–55.07)	51.35 (47.3–55.39)
	T/T	360	13.72 (12.81–14.62)	25.53 (22.48–28.57)	59.53 (55.49–63.57)	6.88 (5.37–8.39)	37.69 (36.06–39.32)	32.94 (30.34–35.54)	17.81 (15.51–20.1)	50.01 (47.36–52.66)	50.04 (47.58–52.5)
*p* value^b^			0.836	0.777	0.411	0.274	0.804	0.285	0.23	0.755	0.501

*Abbreviations*: *CI* confidence interval, *FIQ* Fibromyalgia Impact Questionnaire, *BFI* Brief Fatigue Inventory, *PCS* Physical Component Summary, *MCS* Mental Component Summary, *BDI* Beck Depression Inventory, *STAI-I* State-Trait Anxiety Inventory-I, *STAI-II* State-Trait Anxiety Inventory-II

^a^Missing data were excluded from the analyses: *BDNF* rs2883187 (5 controls), *BDNF* rs7103873 (1 patient and 16 controls), *BDNF* rs7103411 (2 controls), *BDNF* rs10835210 (1 patient and 10 controls), *BDNF* rs11030104 (2 patients and 3 controls), *BDNF* rs12273539 (1 control), *BDNF* rs11030102 (1 patient and 1 control), *BDNF* rs11030101 (1 patient and 3 controls), *BDNF* rs6265 (4 controls and 1 patient), and *BDNF* rs7124442 (2 patients and 2 controls)

^b^*p* values derived by analysis of covariance adjusted for age and sex

### Haplotype frequencies and clinical measurements

Among the 39 haplotype structures included in the haplotype analysis of *BDNF* SNPs, seven frequent haplotypes (TGACCGCTGC, TATCCAACCT, TGACCACTGC, TAACTACCCT, TATCCGACCT, TAACTGCCCT, and CAACCACCGC) had a frequency of > 1% in the patients and controls. Although not shown in Table 4, the total frequency of the other haplotype structures was 30 (3.8%) for patients and 46 (6%) for controls. These haplotypes showed significantly different distributions between the patients with FM and the controls (*P* = 0.0001; Table 4).

**Table 4:** Estimates of haplotype frequencies in patients with fibromyalgia (*n* = 393) and healthy controls (*n* = 388)^a^

Combined alleles^a^	All subjects	Controls	Fibromyalgia	*p* value^b^
TGACCGCTGC	29.6 ± 0.75	20.22 ± 0.9	38.87 ± 0.88	0.0001
TATCCAACCT	20.16 ± 0.44	12.94 ± 0.57	27.29 ± 0.52	
TGACCACTGC	14.8 ± 0.75	25.26 ± 0.89	4.47 ± 0.88	
TAACTACCCT	11.97 ± 0.42	6.99 ± 0.5	16.89 ± 0.53	
TATCCGACCT	8.94 ± 0.45	15.76 ± 0.59	2.21 ± 0.52	
TAACTGCCCT	5.74 ± 0.42	9.8 ± 0.5	1.72 ± 0.53	
CAACCACCGC	3.20 ± 0.17	2.06 ± 0.22	4.33 ± 0.24	

^a^Data are percentages ± SE

^a^Missing data were excluded (*n* = 51). Among 39 haplotype structures, 7 haplotypes with frequency of at least 1% in both the patients and controls are presented

^b^*p* values for permutation test of the null hypothesis that cases and controls are random draws from a common set of haplotype frequencies (number of permutations = 10,000)

Among the frequent haplotypes, the TGACCACTGC haplotype was found less frequently in the patients with FM after adjusting for age and sex (OR 0.004, 95% CI 0.0–0.026, *P* < 0.001; Table 5). Interestingly, the TATCCGACCT and TAACTGCCCT haplotypes were not detected in patients with FM (Table 5) (both *P* > 0.05). In the clinical measures, only anxiety, assessed using the STAI-II score, was significantly different among the patients according to *BDNF* haplotype (Table 6).

**Table 5:** Combined allele frequencies and odds ratios in patients with fibromyalgia and healthy controls^a^

Combined alleles	Controls, *n* (%)	Fibromyalgia, *n* (%)	Crude OR (95% CI)	*p* value^b^	Age and sex adjusted OR (95% CI)	*p* value^b^
TGACCGCTGC	193 (26.6)	340 (45)	1.0 (reference)		1.0 (reference)	
TATCCAACCT	119 (16.4)	232 (30.7)	1.107 (0.834–1.469)	0.483	1.106 (0.833–1.47)	0.487
TGACCACTGC	160 (22)	1 (0.1)	0.004 (0–0.026)	< 0.001	0.004 (0.0–0.026)	< 0.001
TAACTACCCT	66 (9.1)	146 (19.3)	1.256 (0.894–1.765)	0.19	1.248 (0.887–1.756)	0.204
TATCCGACCT	103 (14.2)	0 (0)	0 (0-Inf)	0.963	0 (0-Inf)	0.963
TAACTGCCCT	65 (9)	0 (0)	0 (0-Inf)	0.971	0 (0-Inf)	0.97
CAACCACCGC	20 (2.8)	37 (4.9)	1.05 (0.593–1.861)	0.867	1.088 (0.612–1.934)	0.774

*Abbreviations*: *OR* odds ratio, *CI* confidence interval

^a^Missing data were excluded (n = 51). Among 39 haplotype structures, 7 haplotypes with a frequency of at least 1% in both the patients and controls are presented; the total frequency of the other haplotype structures was 46 (6%) for controls and 30 (3.8%) for patients. Logistic regression models were used to calculate ORs

^b^Computed for the estimated coefficient of each haplotype in the logistic regression

**Table 6:** Numbers of haplotypes and least-squares means (95% CI) of responses in patients with fibromyalgia

Combined alleles	Number^a^	Tender point number	Tender point count	FIQ	BFI	PCS	MCS	BDI	STAI-I	STAI-II
TGACCGCTGC	340	13.61 (12.89–14.33)	25.33 (22.93–27.73)	59.67 (56.48–62.87)	6.81 (5.6–8.01)	37.56 (36.25–38.87)	32.12 (30.06–34.18)	18.02 (16.22–19.83)	50.66 (48.56–52.76)	51.01 (49.07–52.95)
TATCCAACCT	232	13.91 (13.1–14.72)	25.29 (22.6–27.99)	59.88 (56.26–63.5)	6.35 (4.99–7.71)	37.49 (36–38.98)	33.88 (31.54–36.21)	17.25 (15.21–19.3)	49.31 (46.92–51.69)	48.26 (46.07–50.46)
TAACTACCCT	146	13.66 (12.75–14.58)	26.34 (23.29–29.38)	61.2 (57.17–65.23)	7.32 (5.8–8.85)	37.49 (35.84–39.15)	31.73 (29.13–34.33)	19.5 (17.21–21.78)	50.49 (47.83–53.14)	51.16 (48.71–53.6)
CAACCACCGC	37	13.53 (12.02–15.05)	25.96 (20.93–31)	63.17 (56.22–70.11)	5.67 (3.09–8.25)	37.78 (34.92–40.63)	30.7 (26.22–35.18)	20.06 (16.15–23.97)	52.65 (48.1–57.2)	52.74 (48.54–56.93)
*p* value^b^		0.874	0.902	0.696	0.527	0.998	0.271	0.221	0.459	0.027

*Abbreviations*: *CI* confidence interval, *FIQ* Fibromyalgia Impact Questionnaire, *BFI* Brief Fatigue Inventory, *PCS* Physical Component Summary, *MCS* Mental Component Summary, *BDI* Beck Depression Inventory, *STAI-I* State-Trait Anxiety Inventory-I, *STAI-II* State-Trait Anxiety Inventory-II

^a^Missing data were excluded from the analyses

^b^*p* values derived by analysis of covariance adjusted for age and sex

## Discussion

To our knowledge, we were the first to investigate the association between *BDNF* SNPs and FM. We found that the allele and genotype frequencies of *BDNF* rs11030104 were significantly different between the patients with FM and the controls. In comparison, although the allele and genotype frequencies of *BDNF* rs12273539 were not significantly different between the patients with FM and the controls, the TT genotype of *BDNF* rs12273539 was associated with susceptibility to FM. In addition to the individual SNPs, certain *BDNF* haplotypes may be protective against FM or contribute to its symptoms. Therefore, our data imply that *BDNF* gene polymorphisms contribute to the development and symptom severity of FM in the Korean population.

Neurotrophic factors are a family of closely related proteins involved in neuronal survival, growth, and differentiation during development of the nervous system [9]. Neurotrophins comprise four structurally related factors: BDNF, nerve growth factor (NGF), neurotrophin 3 (NT-3), and neurotrophin 4/5 (NT-4/5). Neurotrophins play important roles in the transmission of physiologic and pathologic pain [22]. In particular, BDNF plays key roles in chronic pain conditions. BDNF is synthesized in the DRG, and is transported to the central terminals of the primary afferents in the spinal dorsal horn, where it is involved in the modulation of painful stimuli [9]. BDNF contributes to central sensitization by modulating nociceptive inputs and enhancing hyperalgesia through NMDA-receptor-mediated responses [23]. For these reasons, researchers have been interested in the role of BDNF in chronic pain disorders, including FM [24]. In addition, BDNF plays a role in depressive disorder, which is frequently comorbid with FM; indeed, the serum level of BDNF is altered in patients with depression [25, 26]. Moreover, it can be normalized by antidepressants such as milnacipran [26], which are frequently used in the treatment of FM.

Several clinical studies have evaluated the role of BDNF in the pathogenesis of FM. Patients with FM have increased levels of BDNF in blood [12, 14] and cerebrospinal fluid [27] compared to healthy controls, implying that BDNF is involved in the pathophysiology of FM. In particular, Zanette et al. reported that serum BDNF levels are inversely associated with the pressure-pain threshold in patients with FM [13]. Furthermore, increased serum BDNF mediates the disinhibition of motor cortex excitability and the function of the descending inhibitory pain modulation system in patients with FM [28]. In fact, recent studies have shown that disruptions in default mode network (DMN) connectivity may be associated with impaired pain modulation, leading to the chronic pain seen in FM [29, 30]. Furthermore, certain *BDNF* polymorphisms have an effect on specific aspects of brain function such as DMN connectivity, which is currently considered to be central in the pathogenesis of FM [31]. These findings could be a potential explanation that supports the existence of a mechanistic link between *BDNF* polymorphisms and FM. However, although multiple lines of evidence imply a role for BDNF in the pathogenesis of FM, *BDNF* polymorphisms in these patients have not been investigated extensively.

In this study, we found that certain *BDNF* SNPs are associated with susceptibility to FM. The GG genotype and the G allele of *BDNF* rs11030104 exert a protective effect against FM. In contrast, although the allele and genotype frequencies of *BDNF* rs12273539 did not differ between the patients with FM and controls, the TT genotype of *BDNF* rs12273539 was associated with susceptibility to FM. To date, only one study has evaluated associations between *BDNF* gene polymorphisms and FM. Xiao et al. [32] evaluated whether the *BDNF* Val66Met polymorphism was associated with FM; their results implied that the *BDNF* Val66 Met SNP is associated with a subgroup of patients with FM with high-sensitivity C-reactive protein and high body mass index. Nevertheless, the relative distribution of the *BDNF* Val66Met SNP did not differ between the patients with FM and healthy controls. Similarly, in our study, *BDNF* Val66Val Met was not associated with susceptibility to FM. However, our data demonstrate that other *BDNF* SNPs, such as rs11030104 and rs12273539, were associated with the risk of FM in a Korean population.

Furthermore, our data imply that certain *BDNF* haplotypes exert a protective effect against FM. A haplotype refers to a particular set of closely linked alleles that are inherited as a unit, and haplotype analysis can reveal the pattern of genetic variation associated with certain diseases [33]. Several haplotypes of certain genes are reportedly significantly associated with FM. Diatchenko et al. [34] reported that the ACCG haplotype, which consists of four SNPs (rs6269, rs4633, rs4818, and rs4680) of the catechol-O-methyltransferase (*COMT*) gene, is associated with both FM susceptibility and symptom severity [35, 36]. Similarly, we also suggested that a particular haplotype of *TRPV2* may be associated with susceptibility to FM [37]. In the current study, our findings imply that *BDNF* haplotypes may be involved in the pathophysiology of FM.

Notably, we failed to uncover a direct association between *BDNF* gene polymorphisms and pain-related symptom scales such as the tender point number and count. However, those polymorphisms were related to certain psychological symptoms in patients with FM. In particular, certain *BDNF* SNPs and haplotypes were associated with anxiety symptoms. Since patients with FM have a significantly higher prevalence of anxiety disorders (13–63.8%) [38], our findings imply that *BDNF* gene polymorphisms may indirectly affect FM through their effect on anxiety. However, diverse factors affect the development of FM, including psychological symptoms such as anxiety, so our results should be interpreted carefully.

This study had several limitations. First, it was of a case-control design. Because the purpose of this study was to evaluate the role of *BDNF* SNPs associated with susceptibility to FM, we adopted a target-gene-based approach. Therefore, like the majority of SNP studies, we selected candidate SNPs for a case-control analysis of their association with FM. Second, the multiple tests performed in this study may have increased the type I error. In genetics, controlling for multiple testing is important in estimating thresholds of significance accurately, particularly in genome-wide association studies (GWAS) [39]. However, in this target-gene-based SNP study, we did not consider the potential effects of multiple testing in the analyses. In fact, most published FM SNP case-control studies have not considered the potential effects of multiple testing. Third, we were unable to prospectively evaluate the associations between *BDNF* genetic variation and clinical outcomes. Therefore, further studies are needed to investigate the effect of those genetic polymorphisms on the long-term clinical outcomes of patients with FM. Finally, to overcome the insufficient statistical power, we conducted a large-scale study involving > 800 samples. However, our findings should be replicated in a larger population comprising multiple ethnicities.

## Conclusions

In this study, we evaluated the association between *BDNF* polymorphisms and FM in a large sample of the Korean population. We found that *BDNF* gene polymorphisms influenced susceptibility to FM, and contributed to the severity of certain symptoms of FM. Further evidence from large prospective studies is needed to determine the generalizability of our findings to the broader population and their impact on the clinical outcomes of FM. Moreover, further work is needed to elucidate the biologic and epigenetic mechanisms underlying the complex role of the *BDNF* gene in FM.

## Abbreviations

BDI: Beck Depression Inventory
BDNF: Brain-derived neurotrophic factor
BFI: Brief Fatigue Inventory
CI: Confidence interval
DRG: Dorsal root ganglion
FIQ: Fibromyalgia Impact Questionnaire
FM: Fibromyalgia
ICF: Informed consent form
MCS: Mental Component Summary
NMDA: N-methyl-D-aspartate
NSAIDs: Nonsteroidal anti-inflammatory drugs
PCS: Physical Component Summary
SF-36: 36-Item Medical Outcomes Study Short-Form Health Survey
SNP: Single-nucleotide polymorphism
SNRI: Serotonin-norepinephrine reuptake inhibitors
SSRI: Selective serotonin reuptake inhibitor
STAI-I: State-Trait Anxiety Inventory-I
STAI-II: State-Trait Anxiety Inventory-II
TCA: Tricyclic antidepressant
